# Fetal Growth and Prenatal Exposure to Bisphenol A: The Generation R Study

**DOI:** 10.1289/ehp.1205296

**Published:** 2013-01-03

**Authors:** Claudia A. Snijder, Dick Heederik, Frank H. Pierik, Albert Hofman, Vincent W. Jaddoe, Holger M. Koch, Matthew P. Longnecker, Alex Burdorf

**Affiliations:** 1 The Generation R Study Group, and; 2 Department of Public Health, Erasmus MC, Rotterdam, the Netherlands; 3 Institute of Risk Assessment Sciences, Utrecht University, Utrecht, the Netherlands; 4 Department of Urban Environment and Safety, TNO (Netherlands Organization for Applied Scientific Research), Utrecht, the Netherlands; 5 Department of Epidemiology, and; 6 Department of Pediatrics, Erasmus MC, Rotterdam, the Netherlands; 7 Institute for Prevention and Occupational Medicine, German Social Accident Insurance, Institute of the Ruhr-Universitat, Bochum, Germany (IPA); 8 Epidemiology Branch, National Institute of Environmental Health Sciences, National Institutes of Health, Department of Health and Human Services, Research Triangle Park, North Carolina, USA

**Keywords:** birth weight, bisphenol A, fetal growth, head circumference, pregnancy, urine

## Abstract

Background: Prenatal exposure to bisphenol A (BPA) has been associated with adverse birth outcomes, but findings of previous studies have been inconsistent.

Objective: We investigated the relation of prenatal BPA exposure with intrauterine growth and evaluated the effect of the number of measurements per subject on observed associations.

Methods: This study was embedded in a Dutch population-based prospective cohort study, with urine samples collected during early, mid-, and late pregnancy. The study comprised 219 women, of whom 99 had one measurement, 40 had two measurements, and 80 had three measurements of urinary BPA. Fetal growth characteristics were repeatedly measured by ultrasound during pregnancy and combined with measurements at birth. Linear regression models for repeated measurements of both BPA and fetal growth were used to estimate associations between urinary concentrations of creatinine-based BPA (BPA_CB_) and intrauterine growth.

Results: The relationship between BPA_CB_ and fetal growth was sensitive to the number of BPA measurements per woman. Among 80 women with three BPA measurements, women with BPA_CB_ > 4.22 μg/g crea (creatinine) had lower growth rates for fetal weight and head circumference than did women with BPA_CB_ < 1.54 μg/g crea, with estimated differences in mean values at birth of –683 g (20.3% of mean) and –3.9 cm (11.5% of mean), respectively. When fewer measurements were available per woman, the exposure–response relationship became progressively attenuated and statistically nonsignificant.

Conclusion: Our findings suggest that maternal urinary BPA may impair fetal growth. Because previous studies have shown contradictory findings, further evidence is needed to corroborate these findings in the general population.

Pregnant women are exposed to a variety of chemicals during pregnancy (Woodruff et al. 2011; Ye et al. 2008), which may increase the risk of adverse health outcomes (Stillerman et al. 2008). Environmental exposures that have been associated with adverse fetal development include heavy metals (Llanos and Ronco 2009; Zhu et al. 2010), phthalates (Latini et al. 2006), and pesticides (Gilden et al. 2010; Perera et al. 2005).

Bisphenol A (BPA) is used to make polycarbonate polymers and epoxy resins, along with other raw materials in plastics production, and is present in dental fillings, plastic food and water containers, baby bottles, food wraps, and the lining of beverage and food cans, presenting a large number of opportunities for human exposure (Kuo and Ding 2004; Le et al. 2008; Munguía-López et al. 2005). Given the ubiquity of BPA in the human environment, exposure to BPA is virtually universal (Woodruff et al. 2011). BPA is known to exert estrogenic activity and is considered an endocrine-disrupting chemical (EDC) (Alonso-Magdalena et al. 2012). Concern about EDCs stems from their potential effects via diverse mechanisms, including estrogenic/antiandrogenic properties, antioxidant actions, inhibition of cell cycles, and effects on cell differentiation (Hotchkiss et al. 2008; McLachlan et al. 2006). Some animal studies have shown that exposure to EDCs that mimic sex steroids/steroids affected fetal growth and organ differentiation (Hardin et al. 1981; Kim et al. 2001).

Animal studies have shown that BPA may reduce sperm quality, disturb hormonal balance, and cause reproductive organ damage and malformations, as reviewed by Richter et al. (2007). In rats, different experiments on BPA dosages have presented inconsistent results with both a reduction and a gain in body weight (Kim et al. 2001; Rubin et al. 2001). Recently, several epidemiological studies have considered the potential effects of prenatal exposure to BPA on reproductive health. Miao et al. (2011) reported that the children of 50 mothers with occupational exposure to BPA during pregnancy, ascertained by personal air sampling measurements, had lower birth weight (based on parental report) than 444 children whose parents did not have occupational exposure to BPA. Lee et al. (2008) reported that maternal BPA levels in urine among 125 pregnant women during the first trimester were inversely correlated with fetal head circumference in the third trimester. Wolff et al. (2008) reported that in a study population of 339 women and children, higher urinary BPA concentrations in the third trimester of pregnancy were associated with slightly higher birth weight in offspring, but were not associated with head circumference. However, Philippat et al. (2012) reported positive associations between maternal urinary BPA concentrations and birth weight and head circumference in a study of 191 women and children.

The limited, contradictory findings of epidemiological studies on effects of BPA on fetal weight and birth weight may reflect methodological issues related to exposure assessment. Pharmacokinetic studies suggest that BPA is rapidly metabolized with a short half-life, resulting in low to modest correlations between repeated BPA measurements over 1- to 6-month periods (Nepomnaschy et al. 2009; Teitelbaum et al. 2008). A recent study by Braun et al. (2011) reported an intraclass correlation coefficient of 0.11 for BPA across three repeated urine samples during pregnancy, illustrating the need for repeated urinary BPA measurements during pregnancy to obtain accurate estimates of exposure over time.

With this study we aimed to investigate the effects of prenatal exposure to BPA on intrauterine growth and to evaluate the effects of the measurement strategy chosen on the observed associations.

## Materials and Methods

*Study design*. The Generation R study is a population-based prospective cohort study of growth, development, and health from early fetal life until young adulthood in Rotterdam, the Netherlands (Jaddoe et al. 2010). All pregnant women with an expected delivery date between April 2002 and January 2006 in the study area of Rotterdam were invited to participate. In total, 9,778 pregnant women (a response of 61% among women asked) participated in the study, including 8,880 women who enrolled during pregnancy and another 898 women who enrolled at birth of their child. Extensive assessments were carried out during early pregnancy (gestational age < 18 weeks), mid-pregnancy (gestational age 18–25 weeks), and late pregnancy (gestational age > 25 weeks), including biological samples. The study was approved by the Medical Ethics Committee at Erasmus University Medical Centre, Rotterdam, the Netherlands (MEC 198.782/2001/31). Written informed consent was provided by all participants.

*Urine collection and analysis*. In 2006 among all women who provided one urine sample, a random sample of 100 women was taken and analyzed for organophosphorous pesticide, BPA, and phthalate levels (Ye et al. 2008). In 2010 among all women with multiple urine samples available, a random sample was taken of 120 women, consisting of 40 women with two samples and 80 women with three samples. After exclusion of one twin pregnancy, 219 women with 419 urine samples were available, with 26% of samples from the first trimester, 28% from the second trimester, and 46% from the third trimester of pregnancy. All urine samples (65 mL) were collected between February 2004 and November 2005. All samples were taken between 0800 and 2000 hours in 100-mL polypropylene urine collection containers that were kept maximally 20 hr in a cold room (4°C) before being frozen in 20-mL portions in 25-mL polypropylene vials at –20°C. The urine specimens were analyzed for BPA using tandem mass spectrometry. For the 100 specimens analyzed in 2006, this was done at the Institute of Occupational, Social, and Environmental Medicine of the University of Erlangen, Nurnberg, Germany (Ye et al. 2008). The 120 specimens in 2010 were analyzed at the Institute of Prevention and Occupational Medicine, German Social Accident Insurance, Institute of the Ruhr-Universitat, Bochum, Germany (IPA) (Koch et al. 2012). To determine BPA, analytes were hydrolyzed and separated from 1 mL urine using semiautomated steam distillation and solid-phase extraction. In Erlangen, the limit of detection (LOD) was 0.26 μg/L; at IPA the LOD was 0.05 μg/L. The between-assay coefficient of variation was 8.3% in Erlangen and 5.6% at IPA. The within-assay variability was between 3.4 and 6.5%. In Erlangen, derivatization into *tert*-butyldimethylsilyl was needed; in IPA, due to improvements in measurement method, no derivatization was needed, which minimized the influence of BPA contamination due to sample workup, thus also allowing a lower LOD. Urinary creatinine concentrations were determined by the method described by Larsen (1972).

*Fetal growth and birth outcomes*. We used second- and third-trimester fetal ultrasound measurements with measurements of fetal size at birth to estimate growth rates during pregnancy. We measured growth characteristics to the nearest millimeter using standardized ultrasound procedures in the second (median, 20.5; range, 18.2–25.0 weeks) and third (median, 30.2; range, 27.4–33.8 weeks) trimesters. First-trimester measurements were used to establish gestational age, because use of the last menstrual period for pregnancy dating has several limitations (Verburg et al. 2008b), and most women (76%) in our study population did not know the exact date of their last menstrual period. We used crown–rump length for pregnancy dating until gestational age of 12 weeks, and bi-parietal diameter for pregnancy dating thereafter in all women (Altman and Chitty 1997; Robinson et al. 1979). Estimated fetal weight (EFW) was calculated using the formula by Hadlock et al. (1985). The intraclass correlation coefficient of fetal growth measurements was 0.95, based on 21 subjects, indicating a strong relation for different fetal biometry measurements between and among observers (Verburg et al. 2008a). Internal reference curves based on the total Generation R cohort were made for fetal weight and fetal head circumference during pregnancy, showing typical parabolic patterns. For all growth characteristics in the second and third trimesters, standard deviation scores (SD) were constructed based on distributions in Generation R cohort as a whole (Verburg et al. 2008b). This method closely resembles the commonly used *z*-scores approach suggested by the World Health Organization (World Health Organization 2001). Information about gestational age, sex, weight, length, and head circumference at birth was obtained from medical records and hospital registries. For almost all women (*n* = 217, 99.1%) two measures of fetal growth were available; 157 women (72%) had complete information on all three measurements.

*Potential confounders*. The following well-known determinants of fetal growth were included as covariates in models used to estimate associations between urinary BPA and fetal growth: maternal age, prepregnancy weight, height, educational level, ethnicity, parity, smoking, alcohol use, and folic acid supplement use. Maternal height was measured at intake in the study. Maternal age, educational level, ethnicity, parity, and folic acid supplement use were obtained by questionnaire at enrollment in the study. Maternal smoking habits and alcohol use were assessed by questionnaire in each trimester and classified as abstainer, user until pregnancy was known, or user during pregnancy.

*Statistical analyses*. Values for BPA concentrations below the LOD were imputed as LOD/_√_^–^2. Urinary BPA concentrations were highly skewed and therefore all values were log-transformed (lnBPA_CB_; log-transformed creatinine-based BPA concentration) to obtain normal distributions. Repeated-measures analyses were conducted with the Proc Mixed module of SAS (version 9.2; SAS Institute Inc., Cary NC, USA). First, a mixed-effects model was used with lnBPA_CB_ as the dependent variable to estimate associations between time-independent maternal characteristics and BPA concentrations, taking into account random variation within and between subjects in BPA concentrations. Second, we used mixed-effects models with repeated measurements of fetal head circumference or fetal weight, represented using SD scores, as dependent variables, and continuous lnBPA_CB_ in the previous trimester as the independent variable. Thus, we estimated associations for lnBPA_CB_ in urine samples from the first, second, and third trimester with fetal growth measurements from the second trimester, third trimester, and at birth, respectively.

In addition to modeling lnBPA_CB_ as a continuous variable, we estimated associations with BPA_CB_ categorized into quartiles based on the distribution of BPA concentrations in all 219 women. The final model—for example, for BPA_CB_ as a categorical variable—included gestational age (weeks), three dichotomous indicator variables for the 2nd, 3rd, and 4th quartiles of BPA_CB_ (the lowest category was used as reference category) and interaction terms for gestational age and each BPA_CB_ category, in addition to potential confounders (Snijder et al. 2012). Coefficients for the interaction terms from these models represent the average change in SD score per gestational week associated with higher quartiles of BPA_CB_ exposure relative to the lowest quartile, and can be used to test whether the fetuses of women in the higher quartiles of lnBPA_CB_ concentrations grow at a rate that is significantly different from the fetuses of women in the lowest quartile of lnBPA_CB_.

Missing values for lifestyle and socioeconomic variables were handled by multiple imputations (fully conditional specification, Markov Chain Monte Carlo method) by generating five independent data sets for all analyses, using SPSS version 17.0 for Windows (IBM, Chicago, IL, USA). All variables in Table 1 were included in the imputation procedure.

**Table 1:** Baseline characteristics of all women with at least one available BPA measurement (*n* = 219) participating in the Generation R cohort.

Characteristics	Value
Maternal	
Age at intake (years)	30.8 ± 5.2
Weight before pregnancy (kg) [median (interquartile range)]	63.0 (15.3)
Height measured at intake (cm) [median (interquartile range)]	168.0 (11.0)
Educational level	
Low	39 (17.8)
Mid-low	56 (25.6)
Mid-high	55 (25.1)
High	50 (22.8)
Missing	19 (8.7)
Ethnicity	
Dutch	120 (54.8)
Surinamese and Dutch Antilleans	19 (8.7)
Moroccan and Turkish	29 (13.2)
Other	34 (15.5)
Missing	17 (7.8)
Parity	
Nulliparous	112 (51.1)
Multiparous	99 (45.2)
Missing	8 (3.7)
Smoking	
Yes, during pregnancy	27 (12.3)
Yes, until pregnancy was known	10 (4.6)
No	158 (72.1)
Missing	24 (11.0)
Alcohol	
Yes, during pregnancy	74 (33.8)
Yes, until pregnancy was known	28 (12.8)
No	92 (42.0)
Missing	25 (11.4)
Folic acid use	
No	40 (18.3)
Yes, postconception start	49 (22.4)
Yes, preconception start	82 (37.4)
Missing	48 (21.9)
Birth outcomes	
Gestational age at birth (weeks) [median (interquartile range)]	40 (2.00)
Birth weight (g)	3372.28 ± 589.14
Male sex	105 (47.9)
Head circumference at birth (cm)	33.84 ± 1.49
Length at birth (cm)	50.14 ± 2.17
First-trimester gestational age at urine collection (weeks)	13.24 ± 1.74
Second-trimester gestational age at urine collection (weeks)	20.67 ± 1.12
Third-trimester gestational age at urine collection (weeks)	30.37 ± 1.53
Urine creatinine (g/L) [median (interquartile range)]	0.69 (0.66)
Values are n (%) or mean ± SD for categorical variables unless otherwise indicated. Educational levels were defined as low (primary school, 3 years secondary school), mid-low (> 3 years secondary school, intermediate vocational training), mid-high (higher vocational training, bachelor’s degree), and high (higher academic education).	

The influence of the availability of measurement information on the observed exposure–response relationship was evaluated by comparing three approaches. In the first approach it was assumed that only a single BPA measurement was available per woman. For women with multiple measurements, a random selection procedure was used to assign a single measurement to each woman, resulting in a study sample of 219 urine samples from 219 women to study the association between a single lnBPA_CB_ measurement and measures of fetal growth across pregnancy periods. In the second approach, the study sample was limited to 120 women with at least two lnBPA_CB_ measurements available, with a random selection procedure used to select two measurements for women with three measurements. In the third approach, the study sample was further restricted to the 80 women with three lnBPA_CB_ measurements available across every trimester. For each study sample a similar regression model was used to directly compare exposure-response relationships among all approaches. In a sensitivity analysis we applied these three approaches among the 80 women with complete information, to eliminate potential effects of selective participation in the urine sample procedures that might have biased the analyses with different subsets of women included.

In a second sensitivity analysis, we modeled urine lnBPA concentrations adjusted for creatinine, instead of modeling lnBPA_CB_. Finally, we estimated unlagged associations of urine BPA_CB_ in first-, second-, and third-trimester samples with fetal growth measurements in the same (vs. the previous) trimester. In all statistical analyses a *p*-value of < 0.05 was regarded as statistically significant.

## Results

The mean age of the women at enrollment was 30.8 years (Table 1). Of all women, 22.8% had completed higher education and most were of Dutch origin (54.8%). The majority of women were nulliparous (51.1%). A total of 12.3% of the mothers continued smoking, and 33.8% of the mothers continued drinking alcohol after the pregnancy was known. Compared with the Generation R cohort as a whole, women included in our study were slightly more educated, more often of Dutch origin, and more often multiparous [see Supplemental Material, Table S1 (http://dx.doi.org/10.1289/ehp.1205296)].

There were no significant differences between geometric mean BPA concentrations in the first, second, and third trimester of pregnancy, nor for concentrations according to the analytical laboratory (Erlangen or IPA) [see Supplemental Material, Table S2 (http://dx.doi.org/10.1289/ehp.1205296)]. A lower educational level and Moroccan or Turkish (vs. Dutch) ethnicity was associated with lower lnBPA_CB_, and alcohol use until or during pregnancy was associated with higher lnBPA_CB_ (Table 2).

**Table 2:** Predictors of urinary BPA concentrations in 219 pregnant women participating in the Generation R cohort.

Variables	n	Intercept coefficient	Regression coefficient change in lnBPACB	Percent difference in BPACB
Educational level		1.25		
Low	39		–0.37 (–0.70, –0.03)*	–30.59%*
Mid-low	56		–0.29 (–0.58, 0.00)	–25.11%
Mid-high	55		–0.31 (–0.58, –0.03)*	–26.35%*
High	50		Reference	Reference
Ethnicity		1.05		
Dutch	120		Reference	Reference
Surinamese and Dutch Antilleans	19		–0.00 (–0.38, 0.37)	–0.42%
Moroccan and Turkish	29		–0.43 (–0.79, –0.07)*	–34.87%*
Other	34		0.19 (–0.11, 0.49)	21.41%
Parity		1.10		
Nulliparous	112		Reference	Reference
Multiparous	99		–0.14 (–0.35, 0.07)	–12.80%
Smoking		1.05		
No	158		Reference	Reference
Yes, until pregnancy was known	10		–0.21 (–0.69, 0.28)	–18.72%
Yes, during pregnancy	27		–0.06 (–0.44, 0.31)	–6.17%
Alcohol		0.86		
No	92		Reference	Reference
Yes, until pregnancy was known	28		0.39 (0.06, 0.72)*	47.45%*
Yes, during pregnancy	74		0.22 (–0.02, 0.47)	24.87%
Folic acid supplement use		1.01		
No	40		–0.02 (–0.32, 0.29)	–1.84%
Yes, postconception start	49		0.08 (–0.19, 0.34)	7.80%
Yes, preconception start	82		Reference	Reference
BPACB, creatinine-based BPA concentration. *p < 0.05.				

Table 3 shows the univariable and multivariable linear analyses of BPA_CB_ and fetal weight or fetal head circumference, using all 419 measurements from 219 women. Effect estimates were not statistically significant for BPA_CB_ as a categorical or continuous predictor of fetal weight or fetal head circumference, and adjusting for other factors associated with fetal growth [see Supplemental Material, Table S3 (http://dx.doi.org/10.1289/ehp.1205296)] did not influence the reported associations in Table 3.

**Table 3:** Univariable and multivariable repeated linear regression analyses between prenatal exposure to BPA_CB_ and SD scores of fetal weight and fetal head circumference among 219 pregnant women.

Variable	Fetal weight unadjusted β (95% CI)	Fetal weight adjusted β (95% CI)	Fetal head circumference unadjusted β (95% CI)	Fetal head circumference adjusted β (95% CI)
BPACB (μg/g crea)				
< 1.54	Reference	Reference	Reference	Reference
1.54 < BPACB < 2.51	–0.009 (–0.033, 0.014)	–0.010 (–0.033, 0.014)	–0.016 (–0.045, 0.013)	–0.018 (–0.047, 0.011)
2.51 < BPACB < 4.22	–0.018 (–0.041, 0.006)	–0.015 (–0.038, 0.009)	–0.019 (–0.047, 0.009)	–0.016 (–0.044, 0.013)
> 4.22	–0.001 (–0.024, 0.023)	0.001 (–0.023, 0.025)	–0.018 (–0.049, 0.012)	–0.016 (–0.047, 0.014)
Per unit increase in BPACB	–0.013 (–0.025, –0.001)*	–0.011 (–0.023, 0.002)	–0.005 (–0.020, 0.009)	–0.003 (–0.018, 0.011)
BPACB, creatinine based total BPA concentration. Beta coefficient represents the average decline/increase in SD score of fetal weight or fetal head circumference per gestational week. The adjusted model contains the following covariates: maternal age, educational level, ethnicity, fetal sex, weight before pregnancy, height at intake, smoking during pregnancy, alcohol use during pregnancy, folic acid use, and parity. *p < 0.05.				

When the analysis was restricted to the 80 women with three BPA measurements, growth rates were significantly lower in association with lnBPA_CB_ expressed as continuous variable for both fetal weight and fetal head circumference, but associations did not monotonically decrease with increasing quartiles of exposure (Table 4). When fewer measurements were available per pregnant woman, the exposure–response relationship became progressively attenuated and statistically nonsignificant. The effect estimates of the univariable and multivariable analyses in the restricted study sample were comparable, suggesting little influence of the potential confounders (data not shown).

**Table 4:** Linear regression analyses for repeated measurements of the association between BPA during pregnancy and fetal growth rates according to the number of urine samples analyzed.

Samples/women (µg/g crea)		No. of women	Fetal weight β (95% CI)	Fetal head circumference β (95% CI)
Three samples		80		
BPACB	< 1.54		Reference	Reference
BPACB	1.54 < BPACB < 2.51		–0.041 (–0.081, –0.001)*	–0.052 (–0.098, –0.006)*
BPACB	2.51 < BPACB < 4.22		–0.043 (–0.082, –0.004)*	–0.046 (–0.090, –0.003)*
BPACB	> 4.22		–0.029 (–0.070, 0.012)	–0.066 (–0.113, –0.019)*
BPACB	Per unit increase in BPACB		–0.017 (–0.033, –0.001)*	–0.018 (–0.037, 0.000)**
Two samples		120		
BPACB	< 1.54		Reference	Reference
BPACB	1.54 < BPACB < 2.51		–0.018 (–0.045, 0.009)	–0.018 (–0.055, 0.018)
BPACB	2.51 < BPACB < 4.22		–0.029 (–0.056, –0.003)*	–0.013 (–0.049, 0.022)
BPACB	> 4.22		–0.003 (–0.033, 0.027)	–0.017 (–0.057, 0.023)
BPACB	Per unit increase in BPACB		–0.008 (–0.024, 0.008)	–0.005 (–0.024, 0.013)
One sample		219		
BPACB	< 1.54		Reference	Reference
BPACB	1.54 < BPACB < 2.51		0.003 (–0.027, 0.032)	–0.011 (–0.049, 0.025)
BPACB	2.51 < BPACB < 4.22		0.008 (–0.025, 0.040)	0.003 (–0.036, 0.041)
BPACB	> 4.22		0.025 (–0.002, 0.052)	0.015 (–0.022, 0.051)
BPACB	Per unit increase in BPACB		–0.007 (–0.023, 0.010)	0.011 (–0.008, 0.030)
Abbreviations: BPACB, creatinine-based total BPA concentration; crea, creatinine. Beta coefficient represents the average decrease in SD of fetal weight per gestational week. Adjusted for maternal age, educational level, ethnicity, parity, smoking during pregnancy, alcohol use during pregnancy, height at intake, weight before pregnancy, folic acid supplement use, and sex. *p < 0.05. **p < 0.10.				

**Figure 1:**
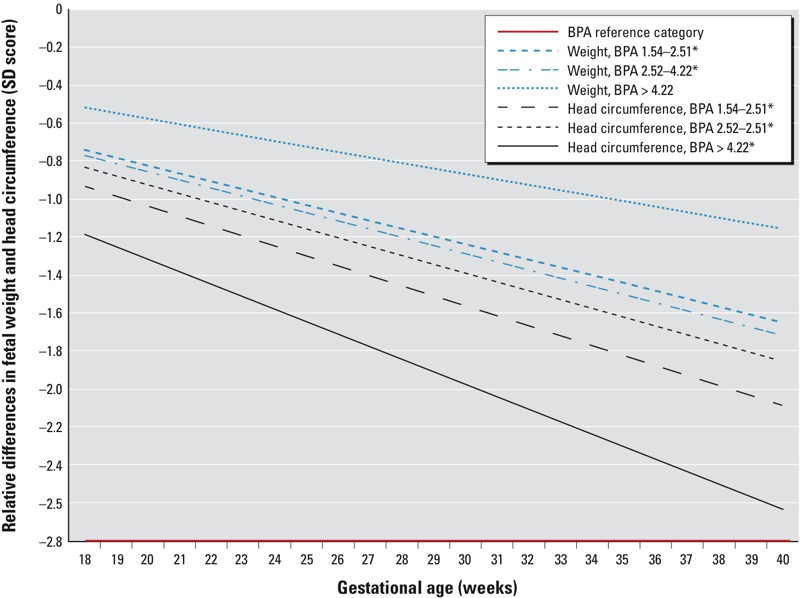
Relative differences in SD scores for fetal weight and head circumference in various BPA_CB_ exposure groups, compared with the lowest (< 1.54) exposure group, among 80 women with urine BPA measurements in each trimester. Adjusted relative differences in fetal weight and head circumference (SD scores) in the highest BPA exposure groups compared with the lowest exposure group. Values are based on linear regression models for repeated measurements and reflect the difference in the SD score of fetal weight or fetal head circumference measurements (based on 238 measurements for fetal weight, and 213 measurements for fetal head circumference) in the offspring of mothers in higher BPA exposure groups compared with the offspring of mothers in the lowest exposure group. The reference value is a SD score of 0. Estimates are adjusted for the following confounders: maternal age, educational level, ethnicity, fetal sex, weight before pregnancy, height at intake, smoking during pregnancy, alcohol use during pregnancy, folic acid use, and parity. *p < 0.05.

In the sensitivity analysis restricted to the 80 women with three BPA measurements, the confidence intervals around the estimates decreased with increasing number of measurements per woman and, in most cases, the magnitude of the association increased [see Supplemental Material, Table S4 (http://dx.doi.org/10.1289/ehp.1205296)]. Specifically, women in the highest BPA exposure group had the lowest estimated growth rates for fetal head circumference, resulting in an average estimated decrease of 2.63 SD at birth, which corresponds to approximately 3.9 cm (11.5%) of the average head circumference of 33.8 cm at birth. For fetal weight, women in the second highest exposure group showed an average estimated decrease of 1.66 SD in birth weight, which corresponds to a difference of 683 grams (20.3%) at birth (Figure 1). Compared with the present study population as a whole, the 80 women with complete urine samples were more likely to be highly educated (33.8% vs. 22.8%) and of Dutch origin (61.3% vs. 54.8%) (data not shown). Effect estimates for fetal growth parameters were comparable for lnBPA_CB_ and lnBPA adjusted for creatinine (data not shown). Effect estimates for first-, second-, and third-trimester BPA concentrations in association with first-, second-, and third-trimester fetal growth parameters (respectively) were very similar to estimates from lagged models (data not shown).

The within- and between-individual variance for lnBPA_CB_ was 1.0728 and 0.4286, respectively, based on 120 women with more than one urine sample.

## Discussion

The findings from this population-based prospective cohort study support our hypothesis that higher concentrations of creatinine-based bisphenol A (BPA_CB_) in prenatal urine may result in lower fetal weight and head circumference. Furthermore, when three BPA measurements were used instead of a single BPA measurement, estimated associations between BPA and fetal growth were stronger and statistically significant. Thus, increasing the number of measurements per subject during pregnancy seems to result in less biased exposure–response estimates.

Epidemiological studies on the effects of prenatal BPA exposure on fetal development are rare. Lee et al. (2008) reported that first-trimester maternal urinary BPA levels in 125 pregnant women were negatively associated with fetal head circumference and abdominal circumference in the third trimester of pregnancy. We also estimated a negative association with head circumference, but a direct comparison of the strength of the exposure–response relationship between studies is not possible. Furthermore, in a study among 587 children from families from which occupational exposure to BPA of 93 fathers and 50 mothers was ascertained through personal air sampling and exposure histories, presence of prenatal occupational exposure to BPA was associated with reduced birth weight, especially for maternal occupational exposure (Miao et al. 2011). Another study among 97 women reported that prenatal BPA exposure, based on concentrations in maternal blood at birth and umbilical cord blood (dichotomized at the median as high or low) were positively associated with low birth weight, small for gestational age, and high adiponectin and low leptin levels in cord blood samples from male, but not female, neonates (Chou et al. 2011). In contrast, Wolff et al. (2008) reported a nonsignificant positive association between urinary BPA concentrations in the third trimester of pregnancy and birth weight in 339 mother–child pairs, but no association with head circumference, and Philippat et al. (2012) reported no associations with birth weight and a monotonic positive associations with head circumference (for the second vs. first tertile of exposure) in 287 mother–child pairs.

Until recently, BPA was considered a weak environmental estrogen, about 10,000–100,000 times less potent than estradiol (Welshons et al. 2003). However, studies on molecular mechanisms have revealed a variety of pathways through which BPA may stimulate cellular responses at very low doses, in addition to higher-dose effects initiated by binding of BPA to the classical or more recent form of the estrogen receptor (Welshons et al. 2006). In humans, BPA is generally described as rapidly metabolized, with elimination thought to be virtually complete within 24 hr after exposure (Philippat et al. 2012). Exposure is thought to be almost exclusively from food; for example, Wilson et al. (2007) estimated that 99% of exposure was of dietary origin, based on BPA measurements from a variety of sources such as food, air, and house dust. However, a recent study by Stahlhut et al. (2009) reported that BPA levels did not decline rapidly with fasting time, which suggests substantial non-food exposure, or accumulation in body tissues such as fat. Braun et al. (2011) observed evidence consistent with numerous sources of BPA exposure during pregnancy, and recommended that epidemiological studies measure BPA concentrations more than once during pregnancy.

Levels of BPA in different trimesters of pregnancy in our study population were similar to levels reported for other study populations. For example, Braun et al. (2011) found GMs of BPA_CB_ of 1.7 (at 16 weeks) and 2.0 (at 26 weeks), whereas geometric mean levels for our population ranged from 1.7 (third-trimester samples analyzed in Erlangen) to 3.3 (second-trimester samples analyzed at IPA).

Our findings suggest that the number of urine samples analyzed may have a profound influence on estimated exposure–response associations. When using all available information on 219 women, no statistically significant associations were observed, whereas associations were stronger based on the analysis restricted to 80 women with three BPA measurements. As mentioned before, women with complete information on BPA were more often of Dutch origin and highly educated—both determinants of higher BPA exposure. Therefore, the restricted study population was more homogeneous for important determinants of exposure, and although the average exposure to BPA was higher, the total variance in BPA was smaller. However, information on co-exposures, such as exposure to other endocrine-disrupting chemicals that may be correlated with BPA [e.g., phthalate concentrations, as noted by Braun et al. (2011)] was lacking.

A second explanation is that any exposure–response association will become attenuated when the exposure varies strongly over time and the most etiologically relevant measure of exposure is the long term average. The attenuation depends on the ratio of the intra- and interindividual variance in exposure, which may be reduced by increasing the number of exposure measurements per subject (Armstrong 1998). Based on a linear regression analysis with repeated exposure measurements and a continuous outcome measure, and the observed ratio of within- over between-person variance of 2.5 for BPA measurements in our study population, we estimate that increasing the number of measurements from 1 to 3 per person would reduce the attenuation from 70% to 45%. The BPA–fetal growth relation may fit the profile of a setting where, in a small study population, more replicates will maximize power more than a proportional increase in number of subjects (Rosner and Willett 1988). This influence of higher random measurement error with fewer measurements per subject available might partly explain the lack of significant findings from some epidemiological of BPA and fetal growth (Philippat et al. 2012; Wolff et al. 2008).

We acknowledge that our study has several limitations, most importantly the small number of women in the analyses, and the limited power to analyze associations with fetal growth of exposure in different time windows. In this study we used ultrasound measurements for pregnancy dating, which is generally expected to be more accurate than dating based on the last menstrual period (Verburg et al. 2008b). However, a disadvantage is that growth variations in early pregnancy are assumed to be zero, impairing analyses of first-trimester growth. The repeated measurements based on gestational age–adjusted SD scores, which are comparable to standardized *z*-scores, enabled us to assess growth restrictions due to pathological factors rather than evaluating those who are constitutionally small. Fetal growth curves during pregnancy have a typical parabolic shape, which can be modeled by using fractional polynomials, but the advantage of SD scores is that growth can be analyzed with a linear model.

A strength of this study is the population-based approach with recruitment during the prenatal period, analysis of multiple urine samples, and adjustment for a large number of potential confounders. Another strength of this study is the multiple observations on fetal growth as well as repeated measurements of BPA per subject. A limitation is the selective participation at baseline, with mothers of lower socioeconomic status less represented in the study population.

Our results suggest that increased concentrations of BPA in urine during pregnancy are associated with a decreased fetal growth for both fetal weight and head circumference. Furthermore, this study supports the use of multiple measurements per subject to quantify exposure in studies on exposure–response relationships. Because previous studies have shown contradictory results and the inherent limitations of our study, we certainly need further evidence before we can conclude that in the general population BPA during pregnancy adversely influences fetal growth.

## Supplemental Material

(610 KB) PDF
