# Supplementary material for: Fetal Growth and Prenatal Exposure to Bisphenol A: The Generation R Study
Source: Environ Health Perspect. 2013 Jan 3;121(3):393–8. doi: 10.1289/ehp.1205296 (PMC3621207; doi:10.1289/ehp.1205296)
Supplement: (610 KB) PDF [file ehp.1205296.s001.pdf]

SUPPLEMENTAL MATERIAL

Manuscript title:

Fetal Growth and Prenatal Exposure to Bisphenol A: The Generation R Study.

Authors:

Claudia A. Snijder, Dick Heederik, Frank H. Pierik, Albert Hofman, Vincent W. Jaddoe,

Holger M. Koch, Matthew P. Longnecker, Alex Burdorf.

Contents:

Supplemental Material, Table S1 Page 2

Supplemental Material, Table S2 Page 3

Supplemental Material, Table S3 Page 4

Supplemental Material, Table S4 Page 5

Supplemental material, Table S1: Characteristics of participants in the BPA fetal growth subset, those with a complete set of pregnancy urine specimens, and the overall Generation R cohort.

Maternal characteristics	BPA fetal growth subset (n=219)	Three urine specimens (n=2083)	Generation R Cohort (n=9778)
**Age at intake (yr)**	30.8 ± 5.2	29.3 ± 5.0	29.9 ± 5.4
**Educational level**			
Low	39 (17.8%)	459 (22.0%)	2270 (23.2%)
Mid-low	56 (25.6%)	591 (28.4%)	2628 (26.9%)
Mid-high	55 (25.1%)	426 (20.5%)	1655 (16.9%)
High	50 (22.8%)	488 (23.4%)	2006 (20.5%)
Missing	19 (8.7%)	119 (5.7%)	1219 (12.5%)
**Ethnicity**			
Dutch	120 (54.8%)	1009 (48.4%)	4443 (45.4%)
Surinamese and Dutch Antilleans	19 (8.7%)	224 (10.8%)	1055 (10.8%)
Moroccan and Turkish	29 (13.2%)	324 (15.6%)	1321 (13.5%)
Other	34 (15.5%)	443 (21.3%)	1931 (19.7%)
Missing	17 (7.8%)	83 (4.0%)	1028 (10.5%)
**Parity**			
Nulliparous	112 (51.1%)	1198 (57.5%)	5179 (53.0%)
Multiparous	99 (45.2%)	867 (41.6%)	4213 (43.1%)
Missing	8 (3.7%)	18 (0.9%)	386 (3.9%)
**Smoking**			
Yes, during pregnancy	27 (12.3%)	283 (13.6%)	1304 (13.3%)
Yes, until pregnancy was known	10 (4.6%)	171 (8.2%)	634 (6.5%)
No	158 (72.1%)	1398 (67.1%)	5656 (57.8%)
Missing	24 (11.0%)	231 (11.1%)	2184 (22.3%)
**Alcohol**			
Yes, during pregnancy	74 (33.8%)	666 (32.0%)	2786 (28.5%)
Yes, until pregnancy was known	28 (12.8%)	311 (14.9%)	1045 (10.7%)
No	92 (42.0%)	895 (43.0%)	3808 (38.9%)
Missing	25 (11.4%)	211 (10.1%)	2139 (21.9%)

Values are means ± standard deviation for normal distributed continuous variables and absolute numbers (percentages) for categorical variables.

Supplemental material, Table S2: Bisphenol A concentrations in three trimesters of pregnancy among 219 women participating in the Generation R cohort

BPA measurements	n (%)	LOD	DF (%)	GM	GSD	Min.	Percentile					Max.
							5th	25th	50th	75th	95th	
N=99 samples analyzed in Erlangen												
Second trimester BPA (µg/l)	13 (13.1%)			1.3	3.5	<lod	<lod	1.0	1.3	3.8	8.0	8.0
Third trimester BPA (µg/l)	86 (86.8%)			1.1	3.9	<lod	<lod	0.5	1.2	2.5	9.5	46.0
Creatinine based second trimester BPA (µg/g Crea)	13 (13.1%)			1.9	2.5	0.4	0.4	1.0	1.9	4.4	8.7	8.7
Creatinine based third trimester BPA (µg/g Crea)	86 (86.8%)			1.7	2.9	0.1	0.3	0.8	1.5	3.9	8.4	22.8
All BPA concentrations (µg/g Crea)	99 (100%)	0.26	84.6	1.7	2.8	0.1	0.3	0.8	1.7	4.0	8.6	22.8
N=120 samples analyzed in Bochum												
First trimester BPA (µg/l)	107 (33.4%)			1.3	3.4	0.1	0.2	0.6	1.1	3.3	10.9	24.6
Second trimester BPA (µg/l)	106 (33.1%)			1.7	3.2	0.2	0.3	0.7	1.7	3.1	13.8	64.8
Third trimester BPA (µg/l)	107 (33.4%)			1.6	3.3	0.2	0.2	0.8	1.5	2.9	17.3	87.2
Creatinine based first trimester BPA (µg/g Crea)	107 (33.4%)			3.1	2.1	0.7	0.9	1.9	3.0	4.9	11.6	23.2
Creatinine based second trimester BPA (µg/g Crea)	106 (33.1%)			3.3	2.3	0.8	1.0	1.9	2.8	4.2	23.4	40.4
Creatinine based third trimester BPA (µg/g Crea)	107 (33.4%)			3.2	2.6	0.6	0.9	1.7	2.5	4.2	34.1	172.0
All BPA concentrations (µg/g Crea)	320 (100%)	0.05	100.0	3.2	2.3	0.6	1.0	1.9	2.8	4.4	14.8	172.0

LOD: limit of detection, DF: detection frequency, GM=geometric mean, GSD=geometric standard deviation, Min: minimum, Max: maximum.

Values below LOD were assigned values LOD/sqrt(2)

Supplemental material, Table S3: Overview of the covariates in the repeated linear regression analyses between prenatal exposure to $lnBPA_{CB}$ and SD scores of fetal weight and fetal head circumference among 219 pregnant women participating in the Generation R Cohort.

Variables	Fetal weight Adjusted beta coefficient (95%CI)	Fetal head circumference Adjusted beta coefficient (95%CI)
Covariates [b]		
Maternal age (per year increase)	0.009 (-0.016, 0.034)	0.014 (-0.014, 0.043)
Pre-pregnancy weight (per kg increase)	0.007 (-0.003, 0.017)	0.004 (-0.007, 0.015)
Height at intake (per cm increase)	0.003 (-0.018, 0.025)	0.007 (-0.016, 0.030)
Gender (female)	0.054 (-0.163, 0.270)	-0.133 (-0.377, 0.112)
Educational level		
Low	0.041 (-0.392, 0.474)	-0.407 (-0.875, 0.062)
Mid-low	-0.115 (-0.438, 0.207)	-0.209 (-0.582, 0.165)
Mid-high	0.013 (-0.279, 0.305)	0.088 (-0.237, 0.413)
High	Reference	Reference
Ethnicity		
Dutch	Reference	Reference
Surinamese and Dutch	-0.627 (-1.063, -0.190)*	-0.178 (-0.645, 0.289)
Antilleans	-0.002 (-0.452, 0.449)	0.093 (-0.420, 0.605)
Moroccan and Turkish	-0.171 (-0.499, 0.158)	-0.359 (-0.723, 0.004)
Other		
Parity	Reference	Reference
Nulliparous	0.311 (0.073, 0.548)*	0.092 (-0.171, 0.354)
Multiparous		
Smoking	Reference	Reference
No	0.083 (-0.396, 0.562)	0.385 (-0.236, 1.006)
Yes, until pregnancy was known	-0.309 (-0.669, 0.051)	-0.021 (-0.433, 0.390)
Yes, during pregnancy		
Alcohol	Reference	Reference
No	0.197 (-0.147, 0.542)	0.036 (-0.350, 0.422)
Yes, until pregnancy was known	0.122 (-0.161, 0.406)	0.051 (-0.252, 0.355)
Yes, during pregnancy		
Folic acid supplement use		
No	-0.241 (-0.609, 0.127)	-0.190 (-0.608, 0.228)
Yes, post conception start	-0.053 (-0.332, 0.227)	0.074 (-0.233, 0.381)
Yes, preconception start	Reference	Reference

[b] beta coefficient is the overall effect of this covariate in the model in SD score of fetal weight or fetal head circumference * P-value <0.05

Supplemental material, Table S4: Linear regression analyses for repeated measurements on the relation between number of urine samples analyzed for BPA per woman and effects on fetal growth rates during pregnancy.

Samples/women		Number of women	Fetal weight Beta coefficient (95%CI)	Fetal head circumference Beta coefficient (95%CI)
Three samples		80		
BPA_{CB} (μg/g Crea)	< 1.54		Ref	Ref
BPA_{CB} (μg/g Crea)	1.54 < BPA_{CB} < 2.51		-0.041 (-0.081, -0.001)*	-0.052 (-0.098, -0.006)*
BPA_{CB} (μg/g Crea)	2.51 < BPA_{CB} < 4.22		-0.043 (-0.082, -0.004)*	-0.046 (-0.090, -0.003)*
BPA_{CB} (μg/g Crea)	> 4.22		-0.029 (-0.070, 0.012)	-0.066 (-0.113, -0.019)*
BPA_{CB} (μg/g Crea)	Per unit increase in BPA_{CB}		-0.017 (-0.033, -0.001)*	-0.018 (-0.037, 0.000)+
Two samples		80		
BPA_{CB} (μg/g Crea)	< 1.54		Ref	Ref
BPA_{CB} (μg/g Crea)	1.54 < BPA_{CB} < 2.51		-0.031 (-0.068, 0.006)	-0.044 (-0.085, -0.002)*
BPA_{CB} (μg/g Crea)	2.51 < BPA_{CB} < 4.22		-0.011 (-0.048, 0.026)	-0.035 (-0.076, 0.005)
BPA_{CB} (μg/g Crea)	> 4.22		-0.006 (-0.044, 0.031)	-0.065 (-0.111, -0.020)*
BPA_{CB} (μg/g Crea)	Per unit increase in BPA_{CB}		-0.016 (-0.036, 0.004)	-0.022 (-0.047, 0.004)+
One sample		80		
BPA_{CB} (μg/g Crea)	< 1.54		Ref	Ref
BPA_{CB} (μg/g Crea)	1.54 < BPA_{CB} < 2.51		-0.007 (-0.057, 0.044)	0.012 (-0.043, 0.068)
BPA_{CB} (μg/g Crea)	2.51 < BPA_{CB} < 4.22		0.015 (-0.035, 0.065)	0.015 (-0.045, 0.075)
BPA_{CB} (μg/g Crea)	> 4.22		-0.030 (-0.081, 0.022)	0.022 (-0.040, 0.085)
BPA_{CB} (μg/g Crea)	Per unit increase in BPA_{CB}		-0.027 (-0.065, 0.010)	0.005 (-0.035, 0.045)

BPA_{CB} = creatinine based total BPA concentration
* P-value <0.05, + P-value <0.10
Beta coefficient represents the average decrease in SD of fetal weight per gestational week.
Adjusted for maternal age, educational level, ethnicity, parity, smoking during pregnancy, alcohol use during pregnancy, height at intake, weight before pregnancy, folic acid supplement use, and gender.
